# Mucormycosis, the Black Fungus in the Post-COVID-19 Pandemic: A Case Report with Review of Literature

**DOI:** 10.7759/cureus.61473

**Published:** 2024-06-01

**Authors:** Aditi M Gandhewar, Alka Hande, Sakshi Akolkar

**Affiliations:** 1 Department of Dentistry, Sharad Pawar Dental College, Datta Meghe Institute of Higher Education and Research, Wardha, IND; 2 Department of Oral Pathology and Microbiology, Sharad Pawar Dental College, Datta Meghe Institute of Higher Education and Research, Wardha, IND

**Keywords:** treatment, india, immunocompromised, covid-19, mucormycosis

## Abstract

Mucormycosis, a concerning and often fatal fungal infection, has shown a significant rise in cases following the COVID-19 pandemic in India, particularly affecting patients with uncontrolled comorbidities such as diabetes mellitus and other immunocompromised individuals. Our case series examines five instances of mucormycosis, supported by appropriate radiographic and histopathological evidence correlating with clinical observations. Our review indicated that patients were experiencing ailments or undergoing treatments that compromised their immune systems. We analyzed additional epidemiological data, including common infection sites, gender predispositions, and mortality rates. Treatments were tailored based on symptom severity, encompassing both surgical and medical approaches. The primary reason for the rise in cases was linked to elevated glycaemic levels and weakened immunity among post-COVID-19 patients. The report provides a detailed explanation of the factors contributing to this correlation. Our findings underscore the critical importance of timely surgical intervention and advocate for further investigation into treatment efficacy and symptom monitoring specific to mucormycosis in post-COVID-19 patients in India.

## Introduction

Mucormycosis, commonly referred to as the "black fungus," is a rare and severe infection that typically proves fatal unless identified early and treated promptly. The time duration before it becomes fatal can vary based on several factors, including the patient's overall health, immune status, the site of infection, and the speed at which treatment is initiated. Its cause is attributed to a fungus from the Mucoraceae family. Rhizopus oryzae is the most common cause of infection among Mucoraceae [1]. Mucormycosis is characterized by the direct infiltration of tissues resulting in necrosis, followed by the progression and invasion of blood vessels. Clinically, mucormycosis can cause nasal blockage, crusting, pain, edema, proptosis, visual defects, headache, pyrexia, and neurological manifestations if cerebral extension occurs. Individuals with compromised immune systems on steroid therapy, uncontrolled diabetes mellitus (DM), and cancer are among the most vulnerable to this fungal infection. There was a dramatic spike in the overall incidence of mucormycosis cases following the COVID-19 pandemic in India. The yearly incidence of mucormycosis is anticipated to vary from 1.7 cases per 1 million people in the US and 140 cases per 1 million cases in India and Pakistan [2]. India is one of the countries with the highest number of cases because of several social determinants of health. Many people lack access to healthcare services, leading to delayed diagnosis and treatment. Comorbidities often remain untreated or uncontrolled because of insufficient education and motivation. Additionally, low socioeconomic status results in unhygienic living conditions and malnutrition, which provide a favorable environment for the fungus to thrive. The infection spreads from the sinuses to the orbit, cavernous sinus, and cranium, and, if not treated, it leads to death, thus with a mortality rate ranging from 25% to 62% [3]. Therefore, early investigation of mucormycosis cases is critical. In this report, we illustrate the diagnostic, therapeutic, and prognostic characteristics of post-COVID-19 mucormycosis.

## Case presentation

Our case report focuses on patients previously admitted to our center, either diagnosed with mucormycosis or found to have the infection after a COVID-19 diagnosis. It encompasses patients admitted from May 2021 onward. The demographic data, comorbidities, HbA1C levels, history of COVID-19, duration between COVID-19 infection and mucormycotic symptoms, use of steroids, clinical findings, evidence of mucormycosis (based on histopathological and radiographic findings), diagnosis, treatments received (both surgical and antifungal therapy), and patient outcomes are presented sequentially in Table 1.

**Table 1:** Case presentation of the patients suffering from mucormycosis. DM, diabetes mellitus; RA, rheumatoid arthritis; HTN, hypertension; DEXA, dexamethasone; MP, methylprednisolone; LAB, liposomal amphotericin B; PCZ, posaconazole; GA, general anesthesia.

Serial number	1	2	3	4	5
Age (in years)	62	48	46	51	52
Sex	Male	Female	Male	Female	Male
Past medical history (comorbidities)	DM	DM	DM+HTN	RA+HTN	DM (incidental finding)
HbA1C	12.4	12.7	13.1	5.6	12
Past medical history of COVID-19	Post-COVID-19	Post-COVID-19	Negative	Negative	Negative
Duration between COVID-19 and symptoms	15 days	17 days	-	-	-
Administration of steroids (dosage and duration)	Yes, DEXA 4 mg (5 days)	Yes, DEXA 6 mg (7 days)	-	Yes, MP 30 mg (25 days)	-
Chief complaints	Pain over the left upper back region of the jaw for the last 15 days.	Pain in teeth on the left side of the upper jaw for the last 15-20 days	Nonhealing lesion over upper left back region of jaw since 20-25 days.	Pain over the upper right back region of the jaw for the last month.	Pain over the upper left region of the jaw for the last 8 days.
Clinical presentation	1. Facial pain. 2. Diffuse reddish pink from 13 to 26 swelling in left maxillary alveolar region. 3. Associated with pus discharge. 4. Reduced mouth opening (20 mm).	1. Facial pain. 2. Hard tender asymmetrical swelling over the left side of the face (6x4 cm). 3. Eroded area seen on hard palate with exposed bone.	1.Diffuse gingival inflammation present wrt 21-28 region. 2. Grade װ mobility seen with 21, 22, 23, 24. 3. Broad bands palpable in buccal mucosa and retromolar region. 4. B/L blanching present over buccal mucosa, labial mucosa, hard and soft palate and retromolar region.	1.Generalised gingival inflammation of maxilla. 2. Sinus tract present with 17. 3. Mobility with 14,16. 4. Pus discharge present.	1. Nasal bleed from left nostril on coughing. 2. Watery left eye. 3. Left side headache. 4. Dehiscence present over left side palatal mucosa. 5. Nerve paresthesia over left cheek. 6. Burning sensation on consumption of spicy food. 7. Difficulty in mastication.
Radiographic evidence	Yes	Yes	Yes	Yes	Yes (soft tissue edematous thickening was seen over the left mandible, maxillary, and orbital region. Thickening of the left buccal mucosa. Right-sided deviation of nasal septum Moderate mucosal collection in left ethmoid and maxillary sinus).
Histopathology (H&E stain)	Presence of nonseptate hyphae and granulation tissue in a specimen of left maxillary sinus lining.	Granulation tissue was seen with many nonseptate hyphae and few branching at right angles in the specimen of left maxillary lining, nasal lining, and right sphenoid sinus lining.	Presence of nonseptate hyphae and few branching at right angles. A few areas of necrosis are also seen in the maxillary bone specimen.	Presence of nonseptate hyphae and few branching at right angles.	Granulation tissue was seen with many nonseptate hyphae and few branching at right angles in the specimen of the maxillary sinus lining, palatal mucosa, and superior nasal turbinates. Moreover, angioinvasion was seen.
Diagnosis	Mucormycosis of left maxilla post-COVID-19 infection	Mucormycosis of maxilla post-COVID-19 infection	Mucormycosis	Mucormycosis	Mucormycosis
Treatment (anti-fungal therapy)	LAB	LAB	LAB	LAB	LAB + Tab PCZ
Treatment (surgical)	Surgical debridement and curettage of left maxilla.	Surgical debridement and curettage of bilateral maxillary, ethmoid, and sphenoid sinuses.	Surgical debridement and curettage of maxilla.	Surgical debridement and curettage of right maxilla.	Surgical debridement and curettage of the left maxilla, maxillary sinus, ethmoid sinus, and sphenoid sinus were done under GA.
Outcome	Discharge	Death	Discharge	Death	Discharge

**Figure 1:**
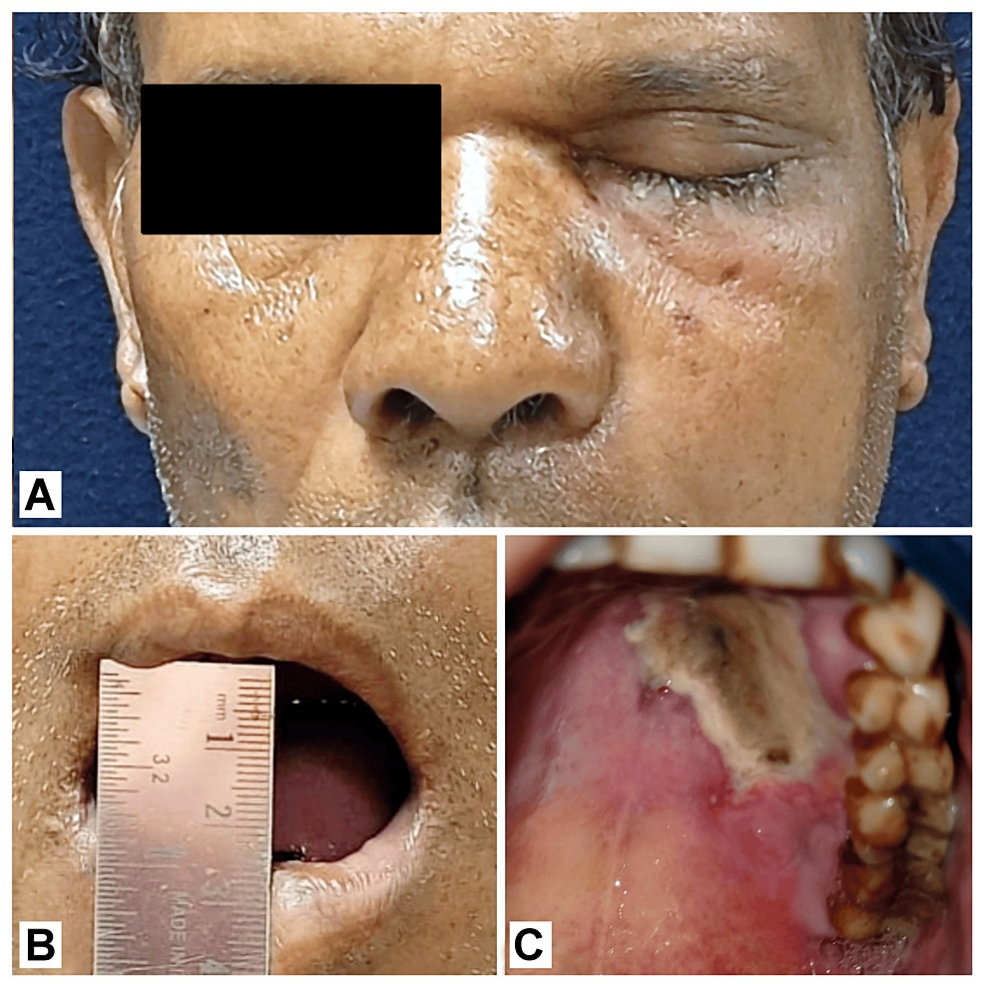
Clinical presentation of mucormycosis of Case 5 showing classical symptoms such as facial swelling, watery eyes (A), reduced mouth opening of about 25 mm (B), and palatal dehiscence (C).

The table provides a comprehensive overview of the case report, highlighting key details such as the age range of the patients (46-62 years) with an average age of 51.8 years. The study showed a male predominance, with 40% female and 60% male patients. Out of the five patients, two had a past medical history of COVID-19, one was diagnosed as diabetic as an incidental finding, and three were known as diabetic, one of whom had concomitant hypertension. We had one patient who suffered from rheumatoid arthritis with hypertension. Except for the patients suffering from rheumatoid arthritis, almost all the patients had increased uncontrollable levels of sugar indicated by HbA1c levels (>8%) [4]. Three of the five patients had received steroids, two were post-COVID-19 patients, and one had rheumatoid arthritis and hypertension (RA + HTN). The average duration of steroid administration was 12.33 days. The most reported symptoms were facial pain, swelling, water eyes (Figure 1A), and dehiscence (Figure 1C). Other typical symptoms included restricted mouth opening (Figure 1B), pus discharge, and teeth mobility.

**Figure 2:**
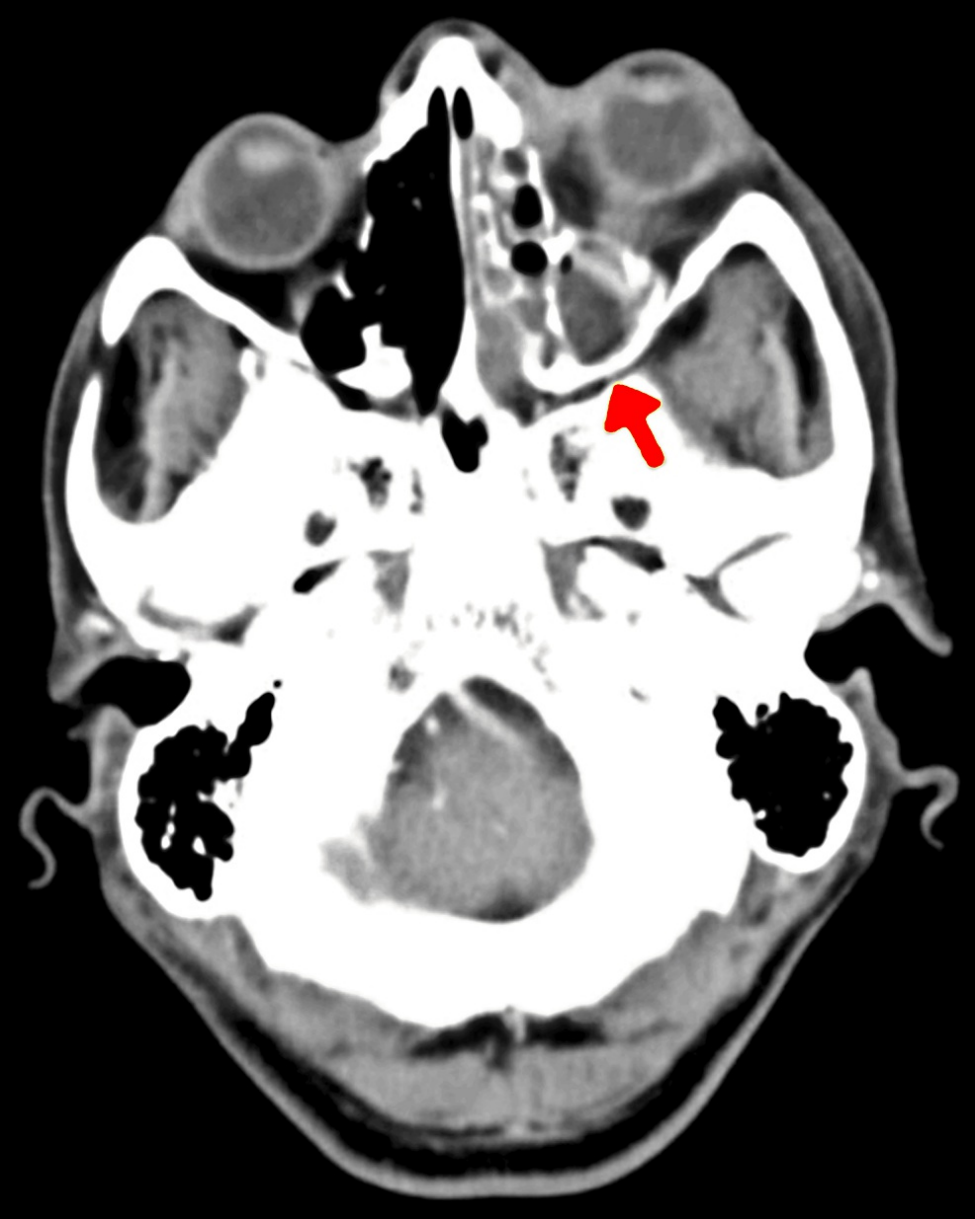
CT face plain of Case 5 shows soft tissue edematous thickening seen over the left mandible, left maxillary, and left orbital region. Right-sided deviation of nasal septum. Moderate mucosal collection in the left ethmoid and left maxillary sinus.

The COVID-19 symptoms lasted an average of 16 days before mucormycosis symptoms emerged. One of two post-COVID-19 patients was admitted to a private hospital in Yavatmal, and the other was admitted to Butibori for COVID-19 treatment. Radiographic evidence of mucormycosis mostly included mucosal collection in sinuses and soft tissue edematous thickenings of the sinuses (Figure 2).

**Figure 3:**
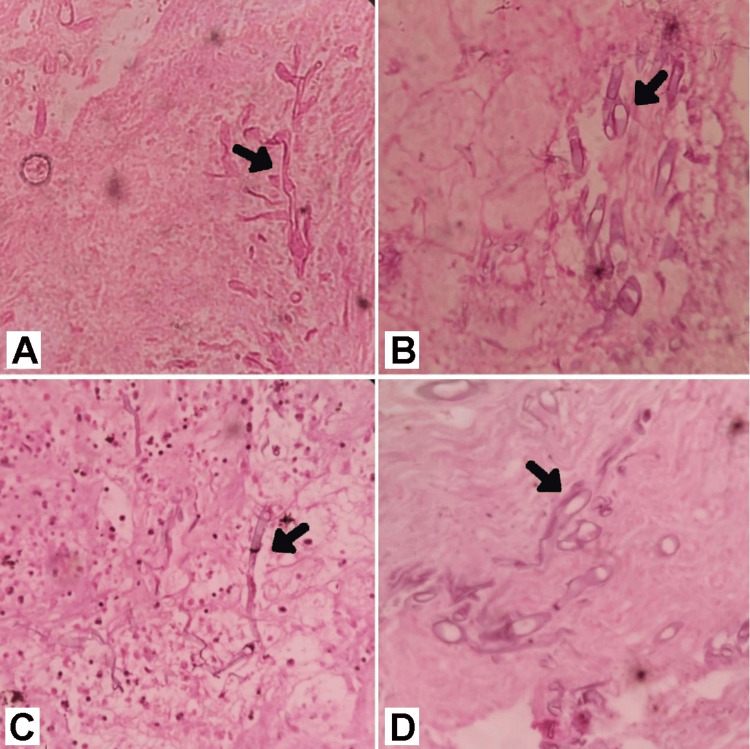
Histological evidence in H&E stain of cases 1(A) in 10X, 2(B) in 40X, 3(C) in 10X, and 4(D) in 40X magnification shows the presence of aseptate fungal hyphae.

**Figure 4:**
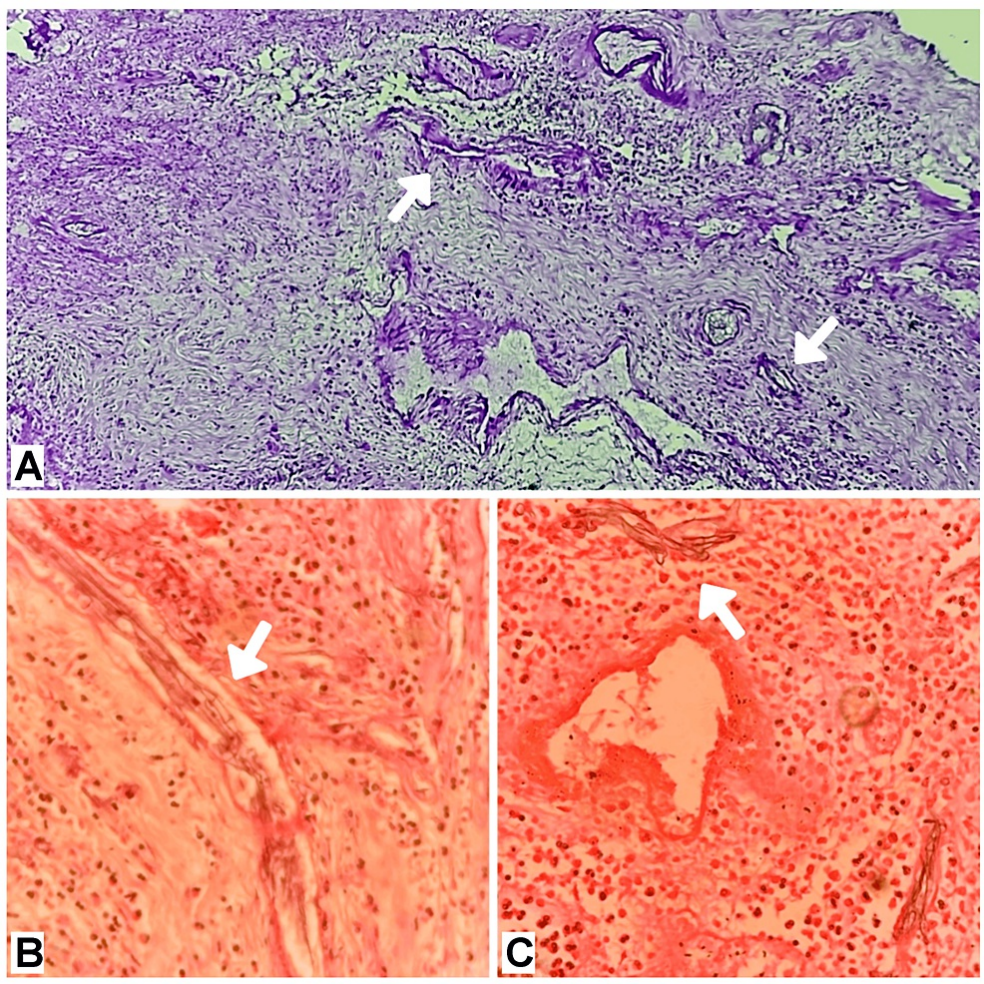
Histological evidence of Case 5 in periodic acid-Schiff (PAS) stain (10X) shows aseptate fungal hyphae(A), in H&E stain (10X) shows characteristic angioinvasion (B) and aseptate fungal hyphae (C).

Histological features such as nonseptate fungal hyphae and few branching at right angles in H&E stains (Figure 3, 4C). In the most recent case of our report (Case 5), we used the periodic acid-Schiff (PAS) stain as well (Figure 4A). In severe cases, histological examination also revealed evidence of angioinvasion (Figure 4B).

Most patients underwent surgical debridement and received treatment with liposomal amphotericin B (LAB) either alone or in conjunction with posaconazole. Two of the five patients died from their illness, while the other three were discharged; hence, the mortality rate was 40%, which is consistent with the other case report findings [3,5].

## Discussion

Mucormycosis is a hazardous fungal infection that targets immunocompromised people with DM, corticosteroid usage, neutropenia, and organ transplantation. The etiological factors include Mucorales and Entomophthorales [5]. The former causes potentially fatal fungal infections, specifically mucormycosis, in immunocompromised hosts, whereas the latter causes cutaneous lesions. Rhizopus oryzae accounts for roughly 90% of the cases.

Phagocytes constitute the primary host immune response against mucormycosis. The use of steroids alters macrophages' ability to suppress the germination of these fungi's spores, and it also impairs leukocyte migration to the site of inflammation by inhibiting cytokines and chemokines [6]. Certain studies show that treatment with dexamethasone at a dose of 6 mg once daily for up to 10 days lowers mortality in COVID-19 patients [7]. However, prolonged usage of glucocorticoids has been shown to increase the patient's susceptibility to infection, particularly opportunistic. As a result, during the COVID-19 pandemic, the incidence of mucormycosis increased, notably in India.

Our data analysis strongly suggests a link between COVID-19 and mucormycotic infestations. The average time between the diagnosis of COVID-19 and mucormycosis was 16 days [8]. Mucormycosis infection is marked by widespread angioinvasion, which leads to vascular thrombosis and tissue destruction, while COVID-19 is characterized by disturbances in the vascular endothelial barrier and reduced oxygen diffusion capacity as in the later stages of coronavirus infection when replication of the virus increases, the epithelial-endothelial barrier is weakened, and the inflammatory response is exacerbated, resulting in an inflow of monocytes and neutrophils. This presents an additional reason for an upsurge in the prevalence of extensive mucormycosis infections in COVID-19 [9].

Other factors, such as unregulated glycemic levels, the usage of industrialized oxygen delivery systems, and the patient's procoagulant condition in COVID-19 are also responsible for the infection. According to recent published studies, COVID-19 promotes coagulation and increases the risk of clot formation. This pro-coagulable state creates an ideal environment for mucor invasion caused by vascular thrombosis [3].

Another reason why India is the country harboring the highest number of mucormycotic patients is that within India solely, probably more than 30 million diabetic patients live. Ketoacidosis is a crucial element leading to the increased tendency of uncontrolled diabetics to Mucor infection as acidic serum reduces the engulfing action of macrophages and the chemotactic action of neutrophils. Furthermore, other serum components, such as the transferring protein, are less efficient at acidic pH levels, allowing free iron to circulate in the blood, which is subsequently consumed by the fungus [10].

The most prevalent clinical finding typically includes pain and swelling, and sinuses were the most prevalent area of mucormycosis among COVID-19 patients at 79.4%, with the maxillary sinus at 47.4% being the most affected [8]. Most of the radiographic evidence of mucor patients such as a CT scan, paranasal sinus X-ray, and magnetic resonance imaging demonstrates sinus mucosal thickening seen in the figure. Histopathological details often comprise the presence of aseptate fungal hyphae, the presence of necrotic tissue, angioinvasion and perineural invasion, and the presence of granulation tissue. The histopathological hallmark suggestive of mucormycosis is the presence of aseptate fungal hyphae and angioinvasion. Significant mortality and morbidity are caused because of the invasive nature of the fungus, which causes blood vessel blockage, leading to widespread tissue death [11].

Patients with mucormycosis react effectively to surgical debridement and proper antifungal medication. International recommendations propose 5-10 mg/kg of LAB every day. Patients must have renal function testing before beginning amphotericin medication [12]. Tablet posaconazole has been demonstrated to be as effective as LAB in treating mucormycosis. The treatment should be followed until both improvement in clinical symptoms and radiographic evidence of disease progression have been resolved. Tablet posaconazole can be used instead of amphotericin B if it is in short supply. Another alternative is to combine amphotericin B and posaconazole, which has equal efficacy but allows for a lower dose of amphotericin B [13].

In cases of rhinocerebral mucormycosis, it is crucial to consider alternative diagnoses such as orbital cellulitis and cavernous sinus thrombosis because of symptom overlap and potential complications [14].

## Conclusions

After analyzing all the cases of mucormycosis in our case report, the following conclusion was made. Successful care of mucormycosis involves early identification, risk factor reduction, antifungal therapy, surgical debridement, and adjuvant therapies such as prosthetic treatment. Implementing prophylaxis measures for high-risk individuals to reduce the incidence of mucormycosis can significantly lower the disease burden in India. While antifungal medications might be an option for prophylaxis, maintaining tight glycemic control in diabetic patients, ensures hygienic living conditions. Adhering strictly to infection control protocols in healthcare settings and educating both healthcare providers and patients about mucormycosis risk factors are crucial steps in reducing its prevalence. The COVID-19 pandemic led to an increase in mucormycosis cases due to steroid use during therapy and the viral infection's impact on patients' overall health. Thus, post-COVID-19 patients should be thoroughly followed for mucormycotic clinical signs. Increased incidence is found in India due to the high prevalence of uncontrolled DM patients (>8% hbA1c level), immunocompromised people, and inadequate healthcare and awareness for the immense population of India. Mucormycosis is most commonly found in the maxillary sinuses, with a male predisposition. Radiographic imaging shows mucosal thickening of sinuses and aseptate hyphae in histopathological slides are seen as the primary signs of the illness. If amphotericin B is in short supply, a combination of amphotericin B and posaconazole can be used instead.
